# Geographical distribution and genetic characterization of *pfhrp2* negative *Plasmodium falciparum* parasites in the Peruvian Amazon

**DOI:** 10.1371/journal.pone.0273872

**Published:** 2022-11-22

**Authors:** Jorge Bendezu, Katherine Torres, Elizabeth Villasis, Sandra Incardona, David Bell, Joseph Vinetz, Dionicia Gamboa

**Affiliations:** 1 Laboratorio de Malaria, Laboratorios de Investigación y Desarrollo, Facultad de Ciencias y Filosofia, Universidad Peruana Cayetano Heredia, Lima, Peru; 2 Escuela Universitaria de Posgrado, Universidad Nacional Federico Villareal, Lima, Peru; 3 Instituto de Medicina Tropical “Alexander von Humboldt” Universidad Peruana Cayetano Heredia, Lima, Peru; 4 Foundation for Innovative New Diagnostics, Geneva, Switzerland; 5 Section of Infectious Diseases, Department of Internal Medicine, Yale School of Medicine, New Haven, CT, United States of America; Johns Hopkins University Bloomberg School of Public Health, UNITED STATES

## Abstract

Malaria rapid diagnostic tests (RDTs) have been evaluated in the Peruvian Amazon region and their performance has been variable. This region is known for being the first with documented evidence of wild *Plasmodium falciparum* parasites lacking *pfhrp2* and *pfhrp3* genes, leading to false-positive results with HRP2-based RDTs. In our attempt to further characterize the deletion pattern of these genes and their evolutionary relationship, 93 *P*. *falciparum* samples, collected in different communities from the Peruvian Amazon region between 2009 and 2010, were analyzed in this study. Genomic DNA was used to amplify 18S rRNA, *pfmsp2* and *pfglurp* to confirm the diagnosis and DNA quality, respectively; *pfhrp2*, *pfhrp3*, and their flanking genes were amplified by PCR to assess the pattern of the gene deletions. In addition, microsatellite analysis were performed using seven neutral microsatellites (MS) and five microsatellite loci flanking *pfhrp2*. The data showed the absence of *pfhrp3* gene in 53.76% (50/93) of the samples, reflecting a higher frequency than the proportion of *pfhrp2* gene deletions (33.33%; 31/93). Among the flanking genes, the highest frequency of deletion was observed in the PF3D7_0831900 gene (78.49%; 73/93) for *pfhrp2*. MS marker analysis showed the presence of 8 *P*. *falciparum* lineages. The lineage Bv1 was the most prevalent among parasites lacking *pfhrp2* and *pfhrp3* genes. Additionally, using MS flanking *pfhrp2* gene, the haplotypes α and δ were found to be the most abundant in this region. This study confirms the presence in this area of field isolates with deletions in either *pfhrp2*, *pfhrp3*, or both genes, along with their respective flanking regions. Our data suggest that some *pfhrp2/pfhrp3* deletion haplotypes, in special the lineage Bv1, are widely dispersed within the Peruvian Amazon. The persistence of these haplotypes ensures a proportion of *P*.*falciparum* parasites lacking the *pfhrp2/pfhrp3* genes in this area, which ultimately leads to false-negative results on PfHRP2-detecting malaria RDTs. However, additional studies are needed to not only confirm this hypothesis but also to further delineate the origin and genetic basis for the *pfhrp2-* and *pfhrp3* gene deletions in wild *P*. *falciparum* parasites.

## Introduction

Early and accurate malaria diagnosis is needed for prompt and appropriate treatment for effective case-management in endemic areas. Accurate diagnosis also reduces unnecessary antimalarial drug usage that can lead to subsequent selection for drug resistant parasites [1, 2]. Laboratory diagnosis based on light microscopy remains the reference standard for malaria diagnosis in Peru [3–5]. However, microscopy is labor-intensive and requires significant resources, equipment and well-trained personnel, which are rarely available in remote areas where most malaria cases occur [3, 4].

Malaria rapid diagnostic tests (RDTs) are the method of choice for filling the diagnostic gap where microscopy is not available. Malaria RDTs use antibodies to bind specific target proteins that are either conserved in all human *Plasmodium* species or specific for one of the species, allowing parasite-based diagnosis to guide treatment. *P*.*falciparum* RDT targets include histidine-rich protein 2 (PfHRP2) and *P*. *falciparum*-specific lactate dehydrogenase (Pf-pLDH); for the other human *Plasmodium* species, targets include pan- or species-specific *Plasmodium* lactate dehydrogenase and aldolase [6, 7]. The performance of malaria RDTs in the field is influenced by many factors like quality of manufacture, handling and storage temperature [8], user interpretation [9], and parasite density [7, 10]. The genetic diversity of the *P*. *falciparum pfhrp2* gene, level of expression, and the protein conformation have been shown to affect the performance of RDTs that detect PfHRP2 [11, 12].

In Peru have been reported a poor performance with HRP2-based RDTs since 2002 [3, 6, 13]. Then a large numbers of *P*. *falciparum* field isolates lacking the *pfhrp2* and/or *pfhrp3* genes were detected for the first time in the Amazon Region [4, 6], North Coast [14] and Central Jungle [15] of Peru. Interestingly, the proportion of parasites with the deletion in *pfhrp3* is high than the proportion of *pfhrp2* deletion [4, 6] however, up to now there is no clear explanation for this event.

The deletion of *pfhrp2* gene was also reported in other malaria endemic countries such as Mali [16], India [17], Ethiopia [18], Nigeria [19], Rwanda [20], Brasil [21, 22], Bolivia [23] and Colombia [24]. Such *pfhrp2*- and *pfhrp3*-negative parasites can lead to false negative RDT results and can therefore affect the performance of PfHRP2-detecting RDTs in these geographical regions. The World Health Organization (WHO) recommends switching to non-HRP2 based alternative RDTs for diagnosis of malaria when the prevalence of false negative results in symptomatic patients caused by confirmed *pfhrp2* deletion exceeds 5% [25].

Initially, *pfhrp2*-deletion was reported in 2010 in samples collected between 2003–2007 from 5 cities (Iquitos, San Lorenzo, Condorcanqui, Jaen and Yurimaguas) of the Peruvian Amazon [4]. A subsequent study by Akinyi S. *et al* [26] utilizing historical samples collected between 1998 to 2005 reported the deletion from 3 collection sites (Caballococha, Padre Cocha and Iquitos). This same study demonstrated that *pfhrp2* deletion was found in multiple clonal lineages suggesting multiple genetic origins of deletion in Peru. Maltha J. *et al* [6] also reported the *pfhrp2* gene deletion in samples collected from four sites within Iquitos: Santa Clara, San Juan, Morona Cocha and Americas. In 2013, Baldeviano GC. *et al* [14] showed evidence of *pfhrp2*-negative parasites in specimens collected in 3 sites (Iquitos, Yurimaguas and Requena), clearly illustrating the widespread distribution over time of these parasites around the Peruvian Amazon.

Between 2009 and 2010, as part of a malaria RDT evaluation programme of the WHO and the Foundation for Innovative New Diagnostics (FIND), blood samples from *P*. *falciparum* positive patients were collected in different sites of the Peruvian Amazon Region (San Juan, Mazan, Atalaya, Urarinas, Requena, Caballococha, Datem del Marañon and Yurimaguas) for extensive molecular characterization using neutral [1] and *pfhrp2* flanking microsatellite markers [26]. The aim was to better understand the genetic structure of these circulating parasites, their geographic distribution in the region, and their impact on test results when PfHRP2-detecting RDTs were used.

## Materials and methods

### Study sites, population and sample collection

A total of 96 *P*. *falciparum* convenient samples were collected from 2009 to 2010 in different sites in the Department of Loreto in the Peruvian Amazon region being Iquitos city the capital of the department located in Maynas province.

One part of these samples (n = 51) were collected during active case detection surveys (ACD), performed by six teams of trained health personnel in 6 different areas where *P*. *falciparum* outbreaks were reported: Datem del Marañon (North-west, border between of the Department of Loreto with Ecuador), Caballococha (East, border between of the Department of Loreto with Brazil and Colombia), Requena-Soplin (South, border between of the Department of Loreto with Brazil), Urarinas (Center of the Department of Loreto, 189 km from Iquitos city), Atalaya (59 km northwest of Iquitos city) and Mazan (38 km at the north-east of the Iquitos city).

The other 45 samples were collected by a Passive Case Detection (PCD) strategy, from patients consulting in health facilities and having a positive *P*. *falciparum* diagnosis, in the San Juan Health Center (4.6 Km from Iquitos city) and the Yurimaguas Hospital (388 km from Iquitos city).

All patients with a positive *P*. *falciparum* diagnosis by microscopy and/or RDT were included if they met the inclusion criteria (patients with confirmed *P*. *falciparum* infection by microscopy or RDT, both sexes, older than 5 years, non-pregnant (in the case of women) and providing written informed consent). Demographic information, clinical symptoms, intake of antimalarial treatment 4 weeks prior to blood sampling and travel history were also recorded. Finger prick blood was collected from *P*. *falciparum*-positive patients onto Whatman™ filter paper (Sigma Aldrich, St, Louis, MO, USA) and FTA cards (GE Lifesciences, MA, USA), dried and sent to the malaria specimen bank at the Laboratory of Malaria at Universidad Peruana Cayetano Heredia (UPCH), Lima, Peru, and the Centers for Disease Control and Prevention (CDC), Atlanta, USA, for species confirmation by 18S rRNA nested PCR and *pfhrp2/pfhrp3* genotyping studies, respectively. After the blood sample was taken, the patient received malaria treatment according to the national guidelines from the Peruvian Ministry of Health [27].

### Malaria microscopy

Malaria microscopy was carried out *in situ* in the two health centers where patients were enrolled by PCD, using the standard microscopy protocols outlined in the Peruvian national guidelines [28]. Two thick and thin smears prepared with a finger-prick sample were stained with 10% Giemsa and examined using a microscope with a 100X oil immersion objective. A second reading was done by a reference microscopist (who receives periodical trainings on malaria diasgnostic and is evaluated by Laboratory of Public Health from Peruvian National Institute of Health (INS) from the Ministry of Health (MINSA)) in a reference center (San Juan Health Center) in Iquitos. The parasite density was calculated as the number of parasites per microliter (par/μl) of blood, by using an average number of 6000 leucocytes per microlitre of blood (according to the Peruvian national guidelines, [28]).

At the ACD sites, where microscopy was not available, patients were diagnosed using malaria RDTs (see below), but thick and thin blood films were also prepared and saved for subsequent staining and analysis at the nearest health center with microscopy capacity. A systematic quality control was performed for all the microscopy slides by two reference microscopists at the reference laboratory (San Juan Health Center) in Iquitos.

### RDTs

Two RDT products were used on both ACD and PCD point of contact: (i) the Advantage Mal Card RDT—Lot number: ACM01079 (J.Mitra &Co. Pvt LTD, Okhla Industrial, New Delhi, India) detecting *P*. *falciparum* specific epitopes of the LDH protein (Pf-pLDH) on one band, and the LDH protein of all *Plasmodium* species on a pan band (pan-pLDH); and (ii) First Response^®^ Combo Malaria Ag (pan-pLDH/HRP2) card test—Lot number: 6960409 (Premier Medical Corporation Limited, Mumbai, Maharashtra, India) based on the detection of PfHRP2 and pan-pLDH. Both RDT types were employed for each patient tested in this study.

### DNA isolation

In the malaria laboratory at UPCH in Lima, parasite DNA was isolated using QIAamp DNA blood mini kit (QIAgen, Hilden, Mettmann, Germany) from 5.0 mm^2^ of blood samples spotted on Whatman^TM^ filter paper, following the manufacturer’s instructions.

At CDC, 2.0 mm^2^ discs of blood spots were cut from each FTA card (Cytiva, MA, USA), the discswere incubated in 200 μl of FTA purification reagent (Sigma Aldrich, Burlington, MA, USA) three times for five minutes each, followed by three five-minute washes with 200μl of TE buffer (10mM Tris-HCl, 0.1mM EDTA, pH 8.0). The discs were then allowed to dry for an hour at room temperature, finally were utilized directly for PCR amplification and microsatellite assays following the manufacter´s instructions [25].

### Species typing and DNA quality

The *Plasmodium* species were confirmed by amplification of the 18S ribosomal RNA gene (18S), and the quality of the *P*. *falciparum* DNA in the samples was assessed by amplification of the *pfmsp2* and *pfglurp* genes, using previously reported procedures [29, 30].

### Genotyping of *pfhrp2*, *pfhrp3 and* flanking genes

At CDC, DNA samples that were confirmed to be positive for *P*. *falciparum* and of good quality (successful amplification of *pfmsp2* and *pfglurp*) were tested for *pfhrp2* (PF3D7_0831800), *pfhrp3* (PF3D7_1372200) and their flanking genes (for *pfhrp2*: PF3D7_0831900 and PF3D7_0831700; and for *pfhrp3*: PF3D7_1372100 and PF3D7_1372400) following a previously reported protocol [26].

### Microsatellite assays

At CDC, *P*. *falciparum* positive and good quality DNA samples were also assayed for seven putatively neutral microsatellites: Ta1, Polyα, PfPk2, TA109, 2490, C2M34 and C3M69 [1], and five microsatellites flanking *pfhrp2* at the following positions: -41kb, 1.4kb, 2.5kb, 5.2kb and 15kb [26]. The amplification products were labeled with fluorescent dyes (HEX or FAM) and assayed for size on an Applied Biosystems 3130xl sequencer. The fragments were then scored with GeneMapper software v.3.7 (Applied Biosystems, Foster City, CA, USA) using default microsatellite settings; bands smaller than 500 relative fluorescence units (rfu) were defined as background according to previous reports using peruvian samples [16, 24]. Samples producing no amplification in some loci were re-analyzed until an amplification were obtained and a clear peak was determinated lineages/haplotypes otherwise samples were excluded from the analysis.

### Data analysis

#### RDTs and microscopy

The sensitivity of both the PfHRP2-detecting RDT and the Pf-LDH detecting RDT was analyzed using PCR 18S as the reference.

#### Deletions of *pfhrp2*, *pfhrp3* and their flanking genes by PCR

PCR assays were performed three times. The PCR products were run on agarose gels for interpretation. When there was a clear positive reaction (a presence of PCR product), the result was accepted with no repetition. When there was a negative test result, the experiments were repeated for confirmation. If results from the first and second PCR were discordant, the experiment was conducted a third time and the final result determined based on the two identical results out of three experiments. The percentage of samples with *pfhrp2* and/or *pfhrp3* deletions was calculated by dividing the number of *pfhrp2* and/or *pfhrp3* PCR negative samples (confirmed to have good quality *P*. *falciparum* DNA with positive *pfmsp2* and *pfglurp* PCR results) divided by the total number of samples successfully amplifying the two control genes.

#### Molecular characterization

*Neutral microsatellites*. To examine the population structure of *P*. *falciparum* isolates, a Bayesian approach was used to infer the number of genetically related clusters (*K mean*) from the individual microsatellite profiles generated using seven neutral microsatellites. Microsatellite analysis was implemented with Structure v2.3.4 [31]. Twenty replicates of the clustering algorithm were performed for each value of *K* with a burn-in period of 10,000 iterations and 100,000 Markov Chain Monte Carlo replications. The admixture model with correlated allele frequencies was also used according to previous reports [26]. Network analysis displaying the phylogenetic relationships between the lineage groups was performed with Network software v.10.0 (Fluxus Technology Ltd, Stanway, CT, England).

*Pfhrp2 flanking microsatellites*. *Pfhrp2* haplotypes were determined by the combination of different alleles detected using microsatellites flanking the *pfhrp2* gene following previously established protocols [26].

#### Ethics statement

The studies involving human participants were reviewed and approved by the Ethical Committee of the Universidad Peruana Cayetano Heredia (Lima, Peru). (SIDISI code 55587). Consent from parents or guardians of the minors were obtained as well as assent from minors’ participants.

## Results

### Patients and samples

Out of a total of 96 samples, two were excluded because the subjects took antimalarial treatment one week before blood collection. One more sample was excluded because it produced a negative result in the PCR targeting the *pfmsp2* gene (see S1 Table). Table 1 shows that nearly half of the 93 samples included in the analysis were collected by PCD from patients consulting in San Juan Health Center and Hospital Santa German in Yurimaguas, while the rest were from patients enrolled by ACD through the surveys carried out in Mazan, Atalaya, Requena, Urarinas, Datem del Marañon and Caballococha. A majority of samples were collected from male participants (56/93, 60.2%). Participants ages ranged from 3 to more than 75 years old.

**Table 1:** Origin of *P*. *falciparum* samples collected in Peruvian amazon (2009–2010).

Study area	Number of samples	Collection system	
**San Juan, Iquitos**	32	PCD	47.31%
**Yurimaguas**	12	PCD	
**Mazan**	11	ACD	52.69%
**Atalaya**	19	ACD	
**Requena**	11	ACD	
**Urarinas**	5	ACD	
**Datem del Marañon**	2	ACD	
**Caballococha**	1	ACD	
**Total**	**93**		**100.0%**

PCD: passive case detection; ACD: active case detection

### Microscopy vs RDTs vs 18S rRNA PCR

Of the 93 *P*. *falciparum* samples included in this study, 92% (86/93) were positive by microscopy, and all 93 samples were positive by 18S rRNA nested PCR, or by at least one type of RDT. When using the 18S rRNA PCR as the reference, the First Response PfHRP2-detecting RDT had a sensitivity of 67.7% compared to 95.7% for the PfLDH-detecting RDT. The PfHRP2-detecting RDT failed to diagnose 30 *P*. *falciparum* samples (false negatives) and 96.6% (29/30) were positives to PfLDH-detecting RDT (see S1 Table).

### Deletions of the *pfhrp2*, *pfhrp3* and flanking genes

Of 93 samples collected in this study, PCR for the *pfhrp2* and the *pfhrp3* genes did not yield the expected PCR product in 33.3% (31/93) and 53.7% (50/93) of the samples, respectively. Also, twenty-two of the 31 *pfhrp2* negative samples (71.0%) were diagnosed as false negative by PfHRP2-detecting RDTs (pan-pLDH/HRP2) considering microscopy (par/ul) as a reference. On the other hand, the remaining 9 samples were true positives for pan-pLDH/HRP2 and had parasitemias ranging from 0 (only gametocytes present) to 31,875 par/μl (see S1 Table).

From the six genes analyzed (PF3D7_0831900, *pfhrp2*, PF3D7_0831700, PF3D7_1372100, *pfhrp3* and PF3D7_1372400), the *pfhrp2* flanking gene called *PF3D7_0831900* was the most frequently absent, in 78.5% of the samples, followed by *pfhrp3* (53.7%) and flanking gene *pfhrp3 –PF3D7_1372100* (38.7%). In addition, both *pfhrp2* and *pfhrp3* genes were deleted in 31.1% of the samples (Table 2).

**Table 2:** Proportion of *pfhrp2*, *pfhrp3* and flanking gene deletions in samples from different sites of the Peruvian Amazon. Data shows the number (and percentage) of parasites with gene deletions out of total samples tested.

Chromosome		8			13		Double deletion
Collection sites (n)	PF3D7_0831900	*pfhrp2-*	PF3D7_0831700	PF3D7_1372100	*pfhrp3-*	PF3D7_1372400	*pfhrp2-/pfhrp3-*
**San Juan (32)**	17 (53.1%)	9 (28.1%)	1 (3.2%)	9 (28.1%)	18 (56.2%)	16 (50.0%)	9 (28.1%)
**Yurimaguas (12)**	12 (100.0%)	2 (16.6%)	2 (16.6%)	2 (16.6%)	4 (33.3%)	1 (8.3%)	1 (8.3%)
**Mazan (11)**	11 (100.0%)	3 (27.2%)	5 (45.4%)	3 (27.2%)	7 (63.3%)	2 (18.1%)	2 (18.1%)
**Atalaya (19)**	16 (84.2%)	0 (0%)	2 (10.52%)	1 (5.2%)	3 (15.7%)	1 (5.2%)	0 (0%)
**Requena (11)**	9 (81.8%)	11(100.0%)	1 (9.0%)	0 (0%)	11 (100.0%)	11 (100.0%)	11 (100.0%)
**Urarinas (5)**	5 (100.0%)	3 (60.0%)	4 (80.0%)	3 (60.0%)	4 (80.0%)	4 (80.0%)	3 (60%)
**Datem del Marañon (2)**	2 (100.0%)	2 (100.0%)	2 (100.0%)	0 (0%)	2 (100.0%)	0 (0%)	2 (100.0%)
**Caballococha (1)**	1 (100.0%)	1 (100.0%)	0 (0%)	0 (0%)	1 (100.0%)	1 (100.0%)	1 (100.0%)
**Total (93)**	**73 (78.5%)**	**31 (33.3%)**	**17 (18.3%)**	**18 (19.4%)**	**50 (53.7%)**	**36 (38.7%)**	**29 (31.1%)**

The deletions of all these genes were widely distributed in samples collected from different sites. None of the 19 samples collected in Atalaya showed *pfhrp2* deletion but 3 samples had *pfhrp3* deletion. All 11 samples collected from Requena showed both *pfhrp2* and *pfhrp3* deletion with some flanking genes intact. The flanking gene *pfhrp3-PF3D7_1372100* was present in all samples collected in Requena and Caballococha (Table 2). The two samples collected in Datem del Marañon were *pfhrp3*-negative, but the flanking genes were present in both samples. The only sample collected in Caballococha was negative for all the genes except the *pfhrp2* flanking gene *PF3D7_0831700*. Double (*pfhrp2* and *pfhrp3*) deletions were identified in one or more samples collected from every collection site except Atalaya.

### *P*. *falciparum* lineages identified using neutral microsatellites

Among the 93 samples used for neutral microsatellite amplification, 88 (94.6%) samples showed positive amplification for the seven microsatellites used. These samples contained alleles related to four genetic lineages A, B, C, and D that were previously reported in Peru [1], and the hybrid lineages C, C/D and B/C were also detected. Interestingly, the allelic profile of the lineage Bv1 [14] was identified in this study.

In this study, we identified a lineage that we named V4, and three additional hybrids between clone A and other previously unreported clones (V1, V2 and V3) (Table 3).

**Table 3:** Neutral microsatellite marker (TA1, Polyα, PfPk2, TA109, 2490, C2M34 and C3M69) profiles for Peruvian samples. The table shows the microsatellite loci sizes obtained for each of the eight haplotypes lineages from samples collected.

Chromosome	6	4	12	6	10	2	3	Reported by
Locus lineage	TA1	Polyα	PfPk2	TA109	2490	C2M34	C3M69	
**A/V1**	172,181*	183	166	160,164*	80	238	134	In this study
**A/V2**	181	182,183**	166	163*,164	79,80*	238	134	
**A/V3**	181	182,183*	166	163,164*	80	239	134	
**V4**	171,172*,178	161,163,164*	175	160	79	232,245*,247	123	
**C**	178	164	163	160	80	246	138	[1]
**C/D**	178	160,161*	175	160*,161	80	232	124	[26]
**B/C**	178	164	172	160*,161	80	225	152	
**Bv1**	171,172*	182*,183	172	160,163*, 164	83	232	133,134*	[15]

* Locus with the major frequency.

**All locus has the same frequency.

**Fig 1:**
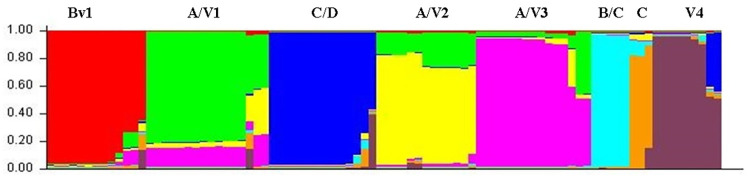
Bayesian cluster analysis of Amazon Peruvian samples. The predicted number of likely clusters (*K*) for samples collected in 2009–2010 (N = 88) was *k* = 8. Each color corresponds to a population classified by Structure v 2.3.4 and each individual isolate is represented by a vertical bar. The Y axis represents the estimated proportion of membership of an individual to each predicted population cluster.

**Fig 2:**
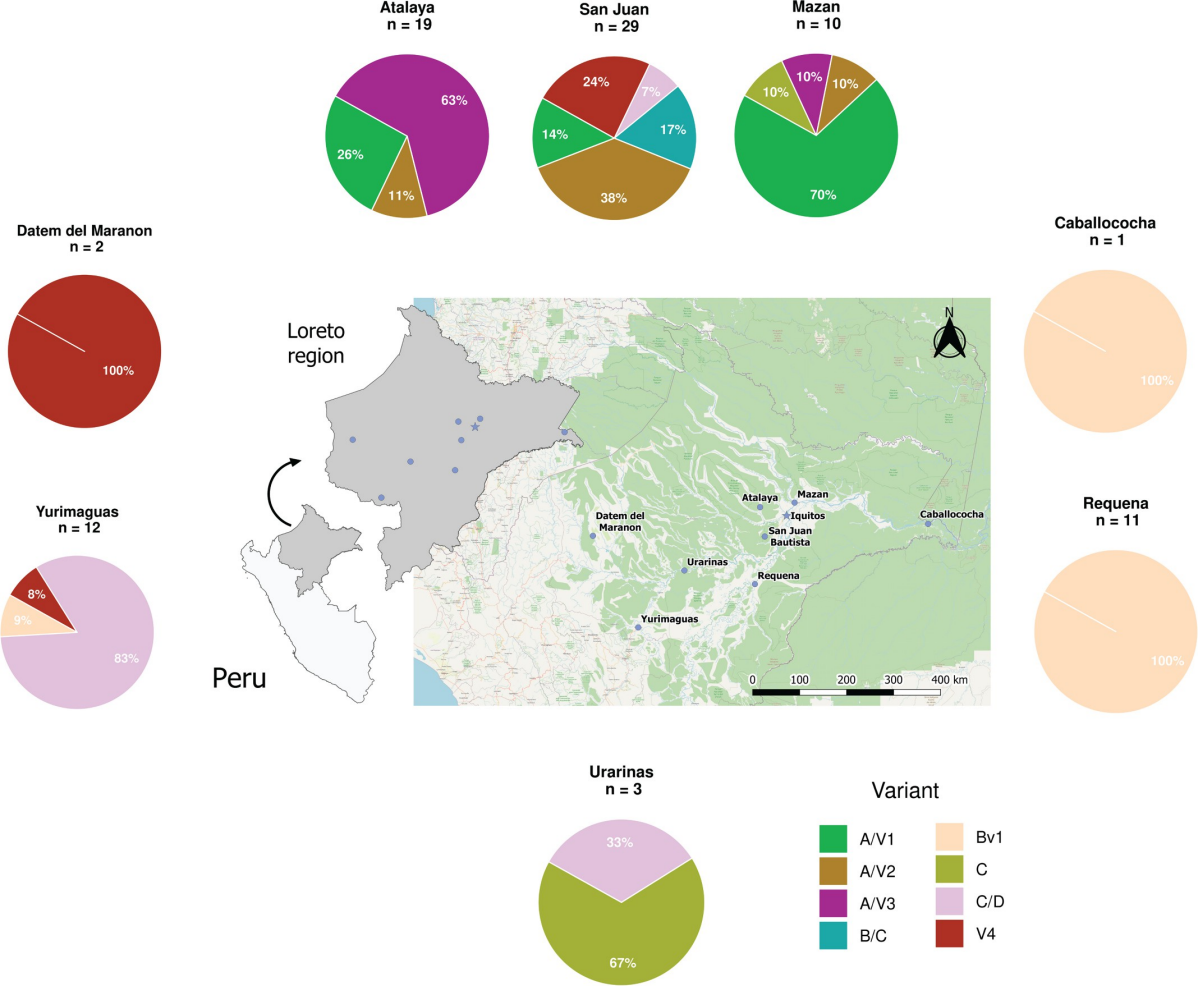
Sample collection sites and lineage distribution in the Peruvian Amazon region. The samples were collected in different areas in the Department of Loreto. Iquitos city, the capital of Loreto is shown by a blue star and the collection sites are shown in blue dots. The proportion of lineages in the study sites are indicated in pie chart and each color represents the lineage identity.

To infer the population structure, a structure analysis was performed on all the samples. The software predicted eight parasite population clusters (*k = 8)* as the best models, therefore we related each lineage with each cluster indentifed by *k-mean* method for further analyses (Fig 1). In total, eight clusters were identified among 88 *P*. *falciparum* DNA samples assayed. Lineage A/V1 was the most frequent since it represented 18.2% (16/88) of the samples. Lineages C and B/C had the lowest frequencies with 3% and 5.7%, respectively (Table 4). The predominant lineage for samples collected in Mazan was A/V1 (70%, 7/10), while A/V2 was predominant in samples collected in San Juan (40.0%, 12/30). 63.1% (12/19) of the Atalaya samples belonged to the A/V3 lineage, while Bv1 was present only in samples collected in Caballococha, Yurimaguas and Requena. Lineage V4 was identified in samples from three sites (San Juan, Yurimaguas and Datem del Marañon). Lineage C was identified in Mazan and Urarinas, while lineage C/D was the most prevalent (83.3%, 10/12) in Yurimaguas. Lastly lineage B/C was only detected in samples collected in San Juan (Fig 2).

**Table 4:** Frequency of lineages identified in the Peruvian collection sites.

Collection Site Lineages	Atalaya	San Juan	Mazan	Caballococha	Requena	Yurimaguas	Datem del Marañon	Urarinas	Total
A/V1	5 (26.3%)	4 (13.3%)	7 (70.0%)	0	0	0	0	0	**16 (18.1%)**
A/V2	2 (10.5%)	12 (40.0%)	1 (10.0%)	0	0	0	0	0	**15 (17.0%)**
A/V3	12 (63.1%)	0	1 (10.0%)	0	0	0	0	0	**13 (14.7%)**
V4	0	7 (23.3%)	0	0	0	1 (8.3%)	2 (100%)	0	**10 (11.3%)**
C	0	0	1 (10.0%)	0	0	0	0	2 (66.6%)	**3 (3.4%)**
C/D	0	2 (6.7%)	0	0	0	10 (83.3%)	0	1 (33.3%)	**13 (14.7%)**
B/C	0	5 (16.6%)	0	0	0	0	0	0	**5 (5.7%)**
Bv1	0	0	0	1 (100%)	11(100%)	1(8.3%)	0	0	**13 (14.7%)**
**Total**	19	30	10	1	11	12	2	3	**88**

**Fig 3:**
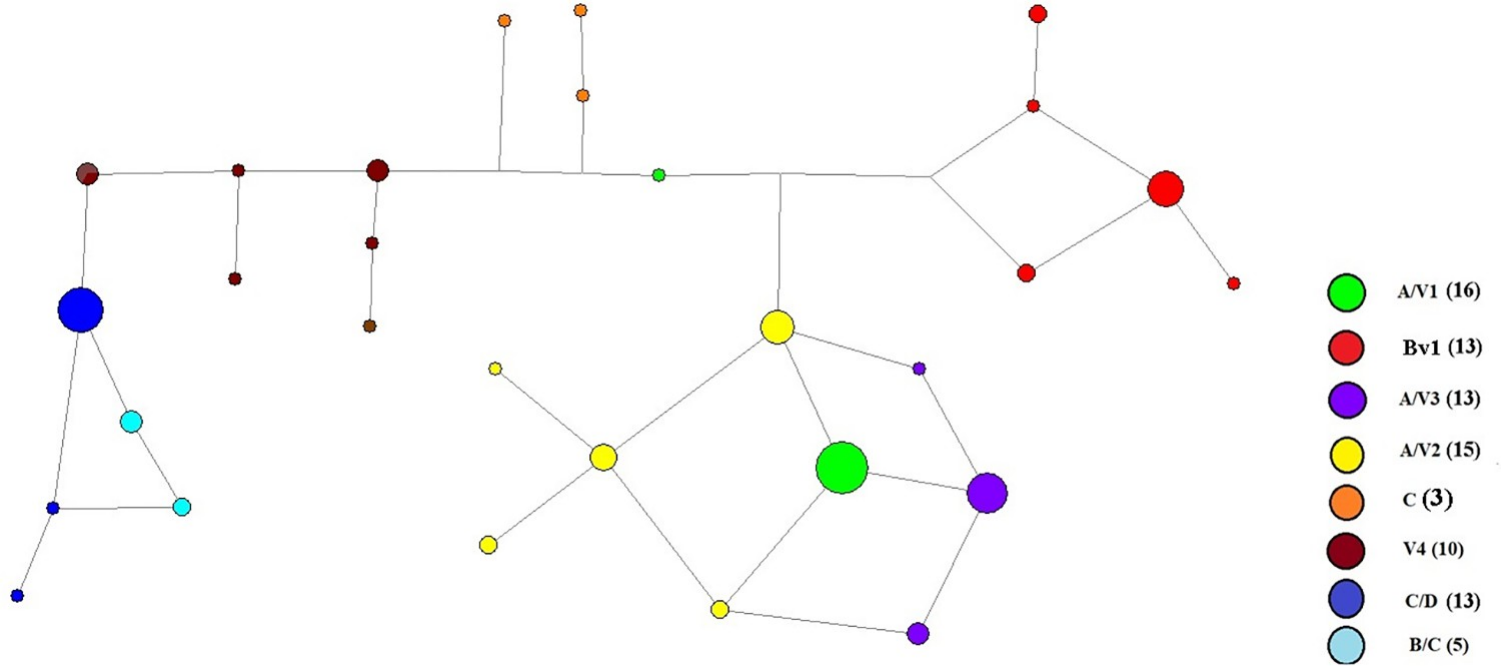
Median joining network analysis of *P*. *falciparum* samples collected in Amazon region in 2009–2010. The genetic relationships among parasites were constructed using the seven neutral microsatellite loci shown on S1 Table. The distinct lineages are rendered in different colors and circle sizes are proportional to the number of samples assigned to a particular lineage. The number of samples assigned to each lineage by Structure analysis are shown in parentheses. The pfhrp2-negative proportions for each lineage are show in bold.

Network analysis and gene flow displaying the phylogenetic relationship among the Peruvian samples (Fig 3). We identified three main groups: the first displays the V4 lineage that shares a genetic background with C/D lineage while the second (lineage Bv1) shows limited genetic diversity on one separated group. The third group demonstrates high genetic diversity among the hybrids A/V1, A/V2 and A/V3.

### Lineages among *pfhrp2*-negative parasites

In the entire sample set analyzed, Bv1 and V4 were not the dominant lineages but were the dominant lineages in gene deleted parasites (*pfhrp2-*, *pfhrp3-* and *pfhrp2-/pfhrp3-*) (Table 5). So, among the *pfhrp2* negative isolates that represented 33.3% (31/93) of all tested samples, 41.9% (13/31) belonged to lineage Bv1 while 32.3% (10/31) belonged to lineage V4. 6.5% (2/31) belonged to lineage A/V1 or AV2 and only one sample belonged to lineage C.

**Table 5:** Proportion of *pfhrp2*, *pfhrp3* and flanking gene deletions in Peruvian samples from different lineage identified. Data shows the number (and percentage) of parasites with gene deletions out of total samples tested.

Chromosome		7			13		Double deletion
Lineages (n)	PF3D7_0831900	*pfhrp2-*	PF3D7_0831700	PF3D7_1372100	*pfhrp3-*	PF3D7_1372400	*pfhrp2-/pfhrp3-*
**A/V1 (16)**	11 (68.7%)	1 (6.2%)	3 (18.7%)	1 (6.2%)	3 (18.7%)	1 (6.2%)	0 (0%)
**A/V2 (15)**	7 (46.6%)	1 (6.6%)	2 (6.7%)	0 (6.7%)	5 (40.0%)	2 (20.0%)	1 (6.7%)
**A/V3 (13)**	10 (76.9%)	0 (0%)	1 (7.69%)	1 (7.69%)	3 (23.7%)	1 (7.69%)	0 (0%)
**V4 (10)**	10 (100.0%)	10 (100.0%)	3 (30.0%)	7 (70.0%)	9 (90.0%)	7 (70.0%)	9 (90.0%)
**C (3)**	3 (100.0%)	1 (33.3%)	1 (33.3%)	1 (33.3%)	3 (100.0%)	2 (66.6%)	1 (33.3%)
**C/D (13)**	13 (100.0%)	0 (0%)	2 (15.4%)	2 (15.4%)	3 (23.1%)	1 (7.69%)	0 (0%)
**B/C (5)**	2 (40.0%)	0 (0%)	0(0%)	0 (0%)	4 (80.0%)	5 (100.0%)	0 (0%)
**Bv1(13)**	11 (84.6%)	13 (100.0%)	1 (7.69%)	0 (0%)	13 (100.0%)	13 (100.0%)	13 (100.0%)

Among the *pfhrp3* negative samples that represented 53.7% (50/93) of all tested samples, 24.0% (13/50) belonged to lineage Bv1 while 22.0% (11/50) belonged to lineage A/V1, AV2 or AV3. On another hand, 18.0% (9/50) belonged to lineage V4 and 6.0% (3/50) belonged to lineage C, while 14.0% (7/50) belonged to lineage B/C or C/D. Parasites lacking both genes (*pfhrp2 and pfhrp3*) belonged to lineage V4 in San Juan (9/10), and lineage Bv1 in Requena (11/11) and Yurimaguas (1/12) (Table 5).

### Identification of *pfhrp2* haplotypes using *pfhrp2* flanking microsatellites

Four *pfhrp2* haplotypes (α, β, γ and δ) were detected in 72/93 studied samples. Haplotype α was predominant (45.2%, 42/93) in samples collected in San Juan, Mazan and Atalaya. The second most common haplotype was δ (23.7%, 22/93) and it was found in samples collected in San Juan, Yurimaguas, Urarinas and Datem del Marañon. Haplotype β was found only in samples from Mazan (6.5%; 6/93) while haplotype γ was present only in two samples from Urarinas (2.2%).

Among the *pfhrp2*-negative isolates, haplotype δ was present in 29.0% (9/31) of the samples from San Juan (7/9) and Datem del Marañon (2/9). Haplotypes α and γ were identified in two isolates from Mazan and one from Urarinas.

Among the *pfhrp3* negative isolates, haplotypes δ (San Juan, Yurimaguas and Datem del Marañon) and α (San Juan, Mazan and Atalaya) were found in 24.0% (12/50) and 22.0% (11/50) of the samples, respectively. Most of the isolates (n = 21) that could not be classified into haplotypes consisted of *pfhrp2* and *pfhrp3* negative parasites, 90.4% (19/21) and 95.2% (20/21) respectively.

**Fig 4:**
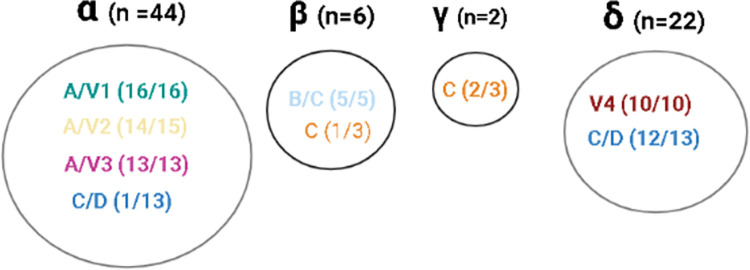
HRP2 haplotype frequency in the Peruvian *P*. *falciparum* samples (n = 74). *P*. *falciparum* samples were included in their respective lineages previously identified in this study and them were grouped (in circles) in new categories called HRP2 haplotypes (α, β, γ and δ) according to HRP2 microsatellite analysis described in Akinyi, S. *et al* 2013. n = indicates the quantity of samples identified for each HRP2 haplotype (inside the circles, the parenthesis indicates the quantity of samples that corresponds to each linages identified using neutral microsattelites).

The four *pfhrp2* haplotypes (α, β, γ and δ) were associated with the eight lineages identified in this study (A/V1, AV/V2, A/V3, V4, C, C/D, B/C and Bv1). The α haplotype was predominant and it was found in A/V1 (16/16), A/V3 (13/13), A/V2 (14/15) and C/D lineages. The second most common haplotype was δ with 22 samples and found in V4 lineage (10/10) and C/D lineage (12/13). The β and γ *pfhrp2* haplotypes were found in a limited number of samples from San Juan, Mazan and Urarinas (n = 8). The β haplotype was detected in all samples belonging to B/C lineage and one sample of the C lineage whileγ haplotype was present in 2/3 samples from the C lineage (Fig 4).

## Discussion

Peru was the first country that reported *P*. *falciparum* field isolates lacking *pfhrp2*, *pfhrp3* and their flanking genes, which have a significant impact on the performance of RDTs based on PfHRP2 detection [4, 6, 32]. In this study, we confirmed the distribution of these parasites in the region based on samples collected from 2009–2010, and we performed molecular characterization using microsatellite markers. These findings show the widespread nature of parasites with *pfhrp2* deletion (33.3%) in this region in 2009–2010, and the most recent studies [2, 15] or sample collections to evaluate RDTs or studies to characterize malaria outbreaks [6] have demonstrated that *pfhrp2* negative parasite populations are expanding in the Peruvian Amazon and other malaria endemic regions in Peru. Therefore, the sole PfHRP2-detecting RDTs are not recommended for *P*. *falciparum* detection in Peru. As has been previously reported, RDTs based on the detection of other antigens like Pf-pLDH and aldolase, as well as quality microscopy, should be used for malaria diagnosis in this area [6, 33].

Interestingly, the deletion of the flanking gene upstream of *pfhrp2* (PF3D7_0831900) was the most prevalent among the Peruvian samples (78.5%, 73/93), in contrast to other regions in the world such as Eritrea, Ethiopia, Brazil, Bolivia or India [16–22, 24]. Today there is no clear evidence about the reason for this deletion, however, a study [26] makes the hypothesis about the relationship between the deletion event of PF3D7_0831900 gene and the emergence of *pfhrp2* deletion. Nonetheless, further genetic assays are necessary to figure out this genetic relation.

Additionally, it is interesting to note that the high frequency of the PF3D7_0831900 deletion is associated with both *pfhrp2* and *pfhrp3* negative parasites. There is no clear reason for the origin of this particular event, however, recently these large genomic deletion in the parasite genome have been attributed to “the telomere healing event” to promote the rapid antigen diversification through mitotic recombination in natural *P*.*falciparum* parasites [34] especially where the telomeric regions are located and their resident genes (PF3D7_0831900, *pfhrp2*, and *pfhpr3*).

### Lineages and *pfhrp2* gene deletion in Peru

Lineages A/V1, A/V2, V4, C/D, and Bv1 were present in three of the study sites, which indicates that they are widespread (Table 4). A high proportion of C/D lineage with no deletions of *pfhrp2* or *pfhrp3* parasites was found in Yurimaguas, however V4, which is the lineage that have a high proportion of the deletions of *pfhrp2* (100%) and *pfhrp3* (90%) genes, has alleles from C/D and C lineages. This might indicate that the *pfhrp2* and *pfhrp3* deletions come as a genetic background from C and C/D lineages. When C/D lineage recombines with lineage B (which was not found in this study), it forms the lineage B/C which has an intact *pfhrp2* gene (0%) but lacks the *pfhrp3* gene (80%), a frequent gene deletion pattern found in the Amazon region [4, 6] and also reported in India [35, 36] and Eritrea [37]. Our results indicate that a recombination process might be occurring among these clones promoting the loss of these genes and possibly others [1].

In Atalaya, most of the samples (16/19) presented no deletion of genes except 3 samples lacking *pfhrp3*. These samples were categorized as lineage A/V3, that is present in Mazan and San Juan along with A/V1 and A/V2, sites where the deletion events are frequent. On the other hand, lineage V4 is present in samples from San Juan—Iquitos that showed double deletion of *pfhrp2/pfhrp3* genes which could be permitting the recombination of these lineages with other parasites circulating with the intact genes such as A/V1 or C/D.

Our results showed that parasites with deletion of *pfhrp2*, *pfhrp3*, or both, are considerably widespread in this part of the region. A recent report suggests that these lineages are highly adaptable to vectors such as *Anopheles darlingi* or *A*. *benarrochi* in comparison with other lineages [14], which could have implications on the expansion and detection of these parasites in other regions of Peru (such as Cusco) [15], but further studies are necessary to understand the real landscape of these lineages in the Peruvian Amazon.

In order to understand the genetic background of the *pfhrp2* gene deletion, we used *pfhrp2* flanking microsatellites. A previous study in Peru [26] established a pattern of haplotypes named α, β, γ, ɛ and δ that had similar geographical distribution to lineages identified using neutral microsatellites in samples collected in 1998–2001; however, the same study using samples from 2003–2005, detected multiple genetic origins for the *pfhrp2* deletion by the identification of six haplotypes (α, β, γ, ɛ, φ and δ)In the present study, the haplotype α associated with A/V1, A/V2 and A/V3 lineages is present in samples with intact genes collected in Mazan and Iquitos while the haplotype δ (V4 and C/D lineages) is presented in San Juan, Datem del Marañon, Urarinas and Yurimaguas where *pfhrp2* deletion samples are detected.The ɛ and φ haplotypes were not identified in the Amazon region during 2009–2010 due to the replacement of circulating lineages by other alleles [14]. Our findings corroborate the hypothesis that geographical dispersion of these linages with multiple genetic backgrounds for the *pfhrp2* deletion can contribute to keeping this genetic characteristic in *P*.*falciparum* parasites population from the Amazon region [26].

Lineage Bv1 is the most geographically dispersed allele since it was found in Requena, Caballococha and Yurimaguas, and it shares a similar genetic background to lineages C and A/V2 (using neutral microsatellites) that correspond to samples with double deletion of *pfhrp2* and *pfhrp3* genes (Table 5 and Fig 3). Lineage Bv1 was previously reported [14] and was associated with two outbreak events in Peru: the first was in the North Coast from 2010–2012 and the second was in the Central Jungle in 2013 [14, 15]. This clone was also described as having *pfhrp2* and *pfhrp3* gene deletions.

### Implications on RDT results

We identified eight samples with a negative result using the PfHRP2-detecting First Response RDT but positive by both *pfhrp2* PCR and the pfLDH-based RDT. Low parasitaemia might explain the outcome for 2 of these samples (≤ 100 parasites /μL). The findings for the other 6 samples might be explained by a different epitope conformation, or low or absent PfHRP2 expression [6, 11]. Maltha J. *et al* [6] similarly observed samples being positive when amplifying the exon 2 (*pfhrp2* gene) but negative with PfHRP2 ELISA assays, another explanation is that most of these samples have a *pfhrp3* intact gene and there are reports about the PfHRP3 cross-reactivity [6, 38].

On the other hand, were identified seven samples (PC-02, PD-01, PD-02, PM-01, PM-08, PS-31 and PU-13) having deleted both *pfhrp2/3* genes gave positive results on the HRP2-detecting RDT (First Response) used. This event was previously reported in Peruvian samples [6] and could be explained by past subclinical infection with PfHRP2 persistence (caused by slow clearance of PfHRP2) [6, 39].

Such types of parasites would limit the application of protocols based on the detection of *pfhrp2/pfhrp3* and their flanking genes as the only assessment tool for predicting testing outcomes with HRP2-based RDTs. Further research is required to better understand and interpret this biological phenomenon.

Until now, it is unclear at which point in time the *pfhrp2*—negative *P*. *falciparum* parasite clones have emerged in this region. However, it has been hypothesized that the low multiplicity of infections and low clonality reported in this region could be a contributing factor [1, 14, 40]. Studies using culture adaptation of these parasites (specially Bv1) could also be useful to identify additional characteristics and to establish their advantages over wild-type parasites.

As a limitation of the present study, we had a reduced number of samples collected in areas like Urarinas, Datem del Marañon, and Caballocoha (Peruvian border with Ecuador, and Brazil and Colombia respectively). Further studies are needed to updated the spatial dispersion of parasites with *pfhrp2* gene deletion from the Amazon region. Future studies need to contemplate the performance of ELISA or other technologies like Luminex for confirming the absence of production of PfHRP2 protein in the blood samples and resolution of discrepant results between RDT, genotyping, and microscopy.

Our study has demonstrated the absence of PCR amplification for *pfhrp2* and *pfhrp3* genes in samples from peri-urban communities around Iquitos city as previously reported [7, 14, 26], but also in other sites farther away from Iquitos city. Even though the number of samples from these remote areas is lower than those from Iquitos city, these results indicate a wide geographical distribution of these lineages (including the previous reported as Bv1) lacking the HRP2 antigen or *pfhrp2* gene in Peru. These results highlight the need for further monitoring studies to map the distribution and extent of these parasite lineages lacking *pfhrp2* and *pfhrp3* genes in other malaria endemic areas in Peru, and throughout South America.

## Supporting information

S1 Table Results by PCR (18S, pfhrp2/pfhrp3 and flanking genes), RDT (first reponse and Advantage Mal Card) and microscopy using 2009–2010 samples (n = 94). (DOCX)
