# Supplementary material for: Geographical distribution and genetic characterization of pfhrp2 negative Plasmodium falciparum parasites in the Peruvian Amazon
Source: PLoS One. 2022 Nov 22;17(11):e0273872. doi: 10.1371/journal.pone.0273872 (PMC9681099; doi:10.1371/journal.pone.0273872)
Supplement: S1 Table — (DOCX) [file pone.0273872.s001.docx]

**Supporting information**

**Table S1**.- Results by PCR (18S, *pfhrp2/3* and flanking genes), RDT (First Reponse and Advantage Mal card) and Microscopy using 2009-2010 samples (n=94).

| **Performated at** | | | **CDC** | | | | | | |  |  | **UPCH** | | | |  |
| --- | --- | --- | --- | --- | --- | --- | --- | --- | --- | --- | --- | --- | --- | --- | --- | --- |
| **Sample ID** | 18S | *pfmsp2* | | PF3D7_0831900 | *pfhrp2* | PF3D7_0831700 | PF3D7_1372100 | *pfhrp3* | PF3D7_1372400 |  |  | *pfglurp* | *First Response* | *Advantaje Mal Card* | *Microscopy(parasites/ul)* | |
| **Atalaya** |  |  | |  |  |  |  |  |  |  |  |  |  |  |  |  |
| PA-01 | + | + | | - | + | + | + | + | + |  |  | + | + | + | 7,673 |  |
| PA-02 | + | + | | - | + | + | + | + | + |  |  | + | + | + | 775 |  |
| PA-03 | + | + | | - | + | + | + | - | + |  |  | + | + | + | 1,773 |  |
| PA-04 | + | + | | - | + | + | + | + | + |  |  | + | + | + | 1,306 |  |
| PA-05 | + | + | | - | + | - | - | - | + |  |  | + | - | + | 42 |  |
| PA-06 | + | + | | - | + | - | + | - | - |  |  | + | - | + | 24 |  |
| PA-07 | + | + | | - | + | + | + | + | + |  |  | + | + | + | 71 |  |
| PA-08 | + | + | | - | + | + | + | + | + |  |  | + | + | + | 4,720 |  |
| PA-09 | + | + | | - | + | + | + | + | + |  |  | + | + | + | 59 |  |
| PA-10 | + | + | | - | + | + | + | + | + |  |  | + | + | + | 1,310 |  |
| PA-11 | + | + | | - | + | + | + | + | + |  |  | + | + | + | 683 |  |
| PA-12 | + | + | | - | + | + | + | + | + |  |  | + | + | + | 47 |  |
| PA-13 | + | + | | - | + | + | + | + | + |  |  | + | + | + | 239 |  |
| PA-14 | + | + | | - | + | + | + | + | + |  |  | + | + | + | 1,200 |  |
| PA-15 | + | + | | - | + | + | + | + | + |  |  | + | + | + | 354 |  |
| PA-16 | + | + | | - | + | + | + | + | + |  |  | + | + | + | 7,477 |  |
| PA-17 | + | + | | + | + | + | + | + | + |  |  | + | - | + | 131 |  |
| PA-18 | + | + | | + | + | + | + | + | + |  |  | + | + | + | 985 |  |
| PA-19 | + | + | | + | + | + | + | + | + |  |  | + | + | + | 2,874 |  |
|  |  |  | |  |  |  |  |  |  |  |  |  |  |  |  |  |
| **Caballococha** | |  | |  |  |  |  |  |  |  |  |  |  |  |  |  |
| PC-02 | + | + | | - | - | + | + | - | - |  |  | + | + | + | 335 |  |
|  |  |  | |  |  |  |  |  |  |  |  |  |  |  |  |  |
| **Datem del Marañon** | | | |  |  |  |  |  |  |  |  |  |  |  |  |  |
| PD-01 | + | + | | - | - | - | + | - | + |  |  | + | + | + | 31,875 |  |
| PD-02 | + | + | | - | - | - | + | - | + |  |  | + | + | + | 28,756 |  |
|  |  |  | |  |  |  |  |  |  |  |  |  |  |  |  |  |
| **Mazan** |  |  | |  |  |  |  |  |  |  |  |  |  |  |  |  |
| PM-01 | + | + | | - | - | - | - | - | - |  |  | + | + | - | *0 |  |
| PM-02 | + | + | | - | + | - | + | - | + |  |  | + | + | + | 175 |  |
| PM-05 | + | + | | - | - | - | - | + | + |  |  | + | + | + | 5,039 |  |
| PM-06 | + | + | | - | + | + | + | + | + |  |  | + | + | + | 355 |  |
| PM-07 | + | + | | - | + | + | + | + | + |  |  | + | + | + | 18 |  |
| PM-08 | + | + | | - | - | - | + | - | + |  |  | + | + | + | 425 |  |
| PM-09 | + | + | | - | + | + | + | - | + |  |  | + | + | + | 4,780 |  |
| PM-10 | + | + | | - | + | + | + | - | + |  |  | + | + | + | 72.5 |  |
| PM-11 | + | + | | - | + | + | + | - | + |  |  | + | + | + | 26 |  |
| PM-12 | + | + | | - | + | + | - | - | - |  |  | + | + | + | 2,946 |  |
| PM-13 | + | + | | - | + | - | + | + | + |  |  | + | - | + | 977 |  |
|  |  |  | |  |  |  |  |  |  |  |  |  |  |  |  |  |
| **Requena** | |  | |  |  |  |  |  |  |  |  |  |  |  |  |  |
| PR-02 | + | + | | - | - | + | + | - | - |  |  | + | - | + | 1,153 |  |
| PR-03 | + | + | | + | - | + | + | - | - |  |  | + | - | + | 254 |  |
| PR-04 | + | + | | - | - | + | + | - | - |  |  | + | - | + | 687 |  |
| PR-05 | + | + | | - | - | + | + | - | - |  |  | + | - | + | 24 |  |
| PR-06 | + | + | | - | - | + | + | - | - |  |  | + | - | + | 2,306 |  |
| PR-07 | + | + | | - | - | + | + | - | - |  |  | + | - | + | 315 |  |
| PR-08 | + | + | | - | - | + | + | - | - |  |  | + | - | + | 355 |  |
| PR-09 | + | + | | - | - | + | + | - | - |  |  | + | - | + | 1,782 |  |
| PR-10 | + | + | | + | - | + | + | - | - |  |  | + | - | + | 205 |  |
| PR-11 | + | + | | - | - | - | + | - | - |  |  | + | - | + | 36 |  |
| PR-12 | + | + | | - | - | + | + | - | - |  |  | + | - | + | 6 |  |
|  |  |  | |  |  |  |  |  |  |  |  |  |  |  |  |  |
| **San Juan** | |  | |  |  |  |  |  |  |  |  |  |  |  |  |  |
| PS-02 | + | + | | + | + | + | + | + | + |  |  | + | + | + | 7,276 |  |
| PS-03 | + | + | | - | + | + | + | + | + |  |  | + | + | + | 4,586 |  |
| PS-04 | + | + | | - | + | + | + | + | - |  |  | + | + | + | 29,282 |  |
| PS-05 | + | + | | + | + | + | + | + | + |  |  | + | + | + | 2,155 |  |
| PS-06 | + | + | | - | + | + | + | + | - |  |  | + | + | + | 25,968 |  |
| PS-07 | + | + | | + | + | + | + | + | + |  |  | + | + | + | 8,432 |  |
| PS-08 | + | + | | - | - | + | - | - | - |  |  | + | - | + | 7,388 |  |
| PS-09 | + | + | | + | + | + | + | - | + |  |  | + | + | + | 3,717 |  |
| PS-10 | + | + | | - | + | + | + | - | - |  |  | + | + | + | 25,668 |  |
| PS-11 | + | + | | + | + | + | + | - | - |  |  | + | + | + | 22,947 |  |
| PS-12 | + | + | | + | + | + | + | - | - |  |  | + | + | + | 36 |  |
| PS-13 | + | + | | - | - | + | + | - | - |  |  | + | - | + | 325 |  |
| PS-14 | + | + | | + | + | + | + | - | - |  |  | + | + | + | 1,145 |  |
| PS-15 | + | + | | + | + | + | + | + | + |  |  | + | + | + | 382 |  |
| PS-16 | + | + | | - | + | + | + | - | + |  |  | + | + | + | 503 |  |
| PS-17 | + | + | | + | + | + | + | - | + |  |  | + | + | + | 7,056 |  |
| PS-18 | + | + | | - | - | + | - | - | - |  |  | + | - | + | 13,938 |  |
| PS-19 | + | + | | + | + | + | + | + | + |  |  | + | + | + | 612 |  |
| PS-20 | + | + | | + | + | + | + | + | + |  |  | + | + | + | 12,791 |  |
| PS-21 | + | + | | - | - | + | - | - | - |  |  | + | - | + | 6,576 |  |
| PS-22 | + | + | | - | - | + | - | - | - |  |  | + | - | + | 5,999 |  |
| PS-23 | + | + | | - | - | + | - | - | - |  |  | + | - | + | 2,823 |  |
| PS-24 | + | + | | - | - | + | - | - | - |  |  | + | - | + | 19,439 |  |
| PS-25 | + | + | | + | + | + | + | + | + |  |  | + | + | + | 580 |  |
| PS-26 | + | + | | + | + | + | + | + | + |  |  | + | + | + | 17,570 |  |
| PS-27 | + | + | | - | + | + | + | + | + |  |  | + | + | + | 2,018 |  |
| PS-28 | + | + | | - | + | + | + | + | + |  |  | + | + | + | 149,025 |  |
| PS-29 | + | + | | + | + | + | + | + | + |  |  | + | + | + | *0 |  |
| PS-30 | + | + | | - | + | + | - | - | - |  |  | + | - | + | *0 |  |
| PS-31 | + | + | | - | - | - | - | - | - |  |  | + | + | - | 21,639 |  |
| PS-32 | + | + | | + | + | + | + | - | + |  |  | + | + | + | 21,639 |  |
| PSJ-01 | + | + | | - | - | + | - | - | - |  |  | + | - | - | 14,067 |  |
|  |  |  | |  |  |  |  |  |  |  |  |  |  |  |  |  |
| **Urarinas** | |  | |  |  |  |  |  |  |  |  |  |  |  |  |  |
| PU-04 | + | + | | - | - | - | + | - | - |  |  | + | - | + | *0 |  |
| PU-05 | + | + | | - | - | - | - | - | - |  |  | + | - | + | *0 |  |
| PU-08 | + | + | | - | + | + | + | - | - |  |  | + | + | + | 4,842 |  |
| &PU-11 | + | - | | - | + | + | - | - | - |  |  | + | - | - | *0 |  |
| PU-13 | + | + | | - | - | - | - | - | - |  |  | + | + | + | *0 |  |
| PU-16 | + | + | | - | + | - | - | + | + |  |  | + | - | + | *0 |  |
|  |  |  | |  |  |  |  |  |  |  |  |  |  |  |  |  |
| **Yurimaguas** | |  | |  |  |  |  |  |  |  |  |  |  |  |  |  |
| PY-003 | + | + | | - | + | - | + | + | + |  |  | + | - | + | 1,480 |  |
| PY-004 | + | + | | - | + | + | + | + | + |  |  | + | + | + | 6,717 |  |
| PY-005 | + | + | | - | + | + | + | + | + |  |  | + | + | + | 27,600 |  |
| PY-006 | + | + | | - | + | + | + | + | + |  |  | + | + | + | 4,821 |  |
| PY-007 | + | + | | - | + | + | + | - | + |  |  | + | + | - | 35 |  |
| PY-008 | + | + | | - | + | + | + | - | + |  |  | + | + | + | 4,929 |  |
| PY-009 | + | + | | - | - | - | - | + | + |  |  | + | + | + | 5,608 |  |
| PY-010 | + | + | | - | + | + | + | + | + |  |  | + | + | + | 1,516 |  |
| PY-011 | + | + | | - | + | + | + | + | + |  |  | + | + | + | 1,500 |  |
| PY-012 | + | + | | - | + | + | + | - | + |  |  | + | + | + | 4,842 |  |
| PY-013 | + | + | | - | - | + | + | - | - |  |  | + | - | + | 15,540 |  |
| PY-014 | + | + | | - | + | + | - | + | + |  |  | + | - | + | 11,852 |  |

& This sample was excluded due the *pfmsp2* negative result.

* This value indicates that throphozoites was not detected.
